# Genome-wide Exploration of a Pyroptosis-Related Long Non-Coding RNA Signature Associated With the Prognosis and Immune Response in Patients With Bladder Cancer

**DOI:** 10.3389/fgene.2022.865204

**Published:** 2022-04-27

**Authors:** Xin Gao, Jianping Cai

**Affiliations:** ^1^ Graduate School of Peking Union Medical College, Chinese Academy of Medical Sciences, Beijing, China; ^2^ The Key Laboratory of Geriatrics, Beijing Institute of Geriatrics, Beijing Hospital, National Center of Gerontology, National Health Commission, Institute of Geriatric Medicine, Chinese Academy of Medical Sciences, Beijing, China; ^3^ Clinical Laboratory, The First People’s Hospital of Huaihua / The Fourth Affiliated Hospital of Jishou University, Huaihua, China

**Keywords:** bladder cancer, pyroptosis, tumor immune microenvironment, lncRNA, prognosis

## Abstract

**Background:** Bladder cancer (BLCA) is a malignant tumor with a complex molecular mechanism and high recurrence rate in the urinary system. Studies have shown that pyroptosis regulates tumor cell proliferation and metastasis and affects the prognosis of cancer patients. However, the role of pyroptosis-related (PR) genes or long non-coding RNAs (lncRNAs) in BLCA development is not fully understood.

**Methods:** We comprehensively analyzed the molecular biological characteristics of PR genes in BLCA, including copy number variation, mutations, expression and prognostic value based on TCGA database. We then identified PR lncRNAs with prognostic value based on the expression of PR genes and performed a consistent clustering analysis of 407 BLCA patients according to the expression of prognosis-related PR lncRNAs and identified two clusters. The least absolute shrinkage and selection operator (LASSO) regression was used to establish a PR lncRNA signature and calculate the risk score associated with the prognosis of patients with BLCA. Gene Ontology (GO), Kyoto Encyclopedia of Genes and Genomes (KEGG) and Gene Set Enrichment Analysis (GSEA) were used to evaluate the possible functions of PR lncRNA signature. We also evaluated the relationship between the risk score and tumor immune microenvironment (TIME).

**Results:** A total of 33 PR genes were obtained in our study and 194 prognosis-related PR lncRNAs were identified. We also constructed a signature consisting of eight-PR-lncRNAs and divided patients into high- and low-risk groups. The overall survival rate of patients with a high risk was significantly lower than patients with a low risk. The risk score was significantly correlated with the degree of infiltration of multiple immune cell subtypes and positively correlated with multiple immune checkpoint genes expression in BLCA. Enrichment analyses showed that these lncRNAs are involved in human immune regulatory functions and immune-related pathways.

**Conclusion:** Our study comprehensively studied the molecular biological characteristics of PR genes BLCA, and the eight-PR-lncRNA signature we identified might play a crucial role in tumor immunity and may be able to predict the prognosis of BLCA patients, providing a theoretical basis for an in-depth study of the relationship between the prognosis and TIME.

## Introduction

Bladder cancer (BLCA) is the second-most common cause of death from urological tumors, and the incidence is still on the rise (Siegel et al., 2019). Non-muscle-invasive bladder cancer (NMIBC) accounts for about 75% of all primary bladder cancers. Unfortunately, 25% of cases have already developed into muscle-invasive bladder cancer (MIBC) by the time of the initial diagnosis (Kaufman et al., 2009). According to the pathological characteristics of BLCA patients, the main clinical treatments are surgery, radiotherapy, chemotherapy, bladder irrigation therapy and combination therapy (Ghandour et al., 2019). However, 10–30% of patients with NMIBC progress to MIBC after recurrence (Malmström et al., 2017), which has a high risk of metastasis and a poor prognosis, with only a minority of patients surviving more than 5 years (Chou et al., 2016). Immunotherapy is an emerging approach to oncology. Immune checkpoint inhibitors (ICIs) effectively block the escape of cancer cells from immune system surveillance, and these agents have begun to change the treatment strategy for BLCA. Recent studies have shown that infiltration of different immune cells may affect the response to ICIs (Benitez et al., 2020). Moreover, long non-coding RNAs (lncRNAs) are closely related to the effect of immunotherapy in BLCA, e.g., knockdown of lncRNA UCA1 significantly enhances the effect of immune checkpoint PD-1 blockers (Zhen et al., 2018). BLCA has a complex molecular biological mechanism, which is one of the main reasons for the poor efficacy of most therapies, lncRNA plays an important biological role in the progression, cell proliferation and metastasis of BLCA, for example, LINC00958 can promote BLCA by targeting miR-490-3p and AURKA (Zhen et al., 2021). lncRNAs can also act as competitive endogenous RNAs (ceRNAs) targeting snuclear factor-kappaB (NF-κB)-activated miRNAs to promote tumor development (Mirzaei et al., 2021). In addition, lncRNAs are involved in BLCA drug resistance and progression through various pathways (e.g. NF-κB, PI3K/Akt, Wnt, FOXC2 and EZH2), which has important implications for the treatment and prognosis of BLCA patients (Barth et al., 2020; Ashrafizaveh et al., 2021; Mirzaei et al., 2022). Therefore, the important role of lncRNA in BLCA has also been gradually emphasized in recent years. The development of genome sequencing and bioinformatics can help identify many molecular biomarkers to guide the treatment of BLCA patients, but only a few of these can be applied in a clinical setting (Zhang et al., 2021). Therefore, identifying the drivers and inhibitors of bladder carcinogenesis and understanding their mechanisms are essential for detecting new therapeutic targets and prolonging the survival of BLCA patients.

Pyroptosis, also known as inflammatory necrosis, is a form of programmed cell death involving cellular swelling until the cell membrane ruptures. The release of cellular contents leads to an intense inflammatory response (Loveless et al., 2021). Pyroptosis is also an essential part of the body’s natural immune response and plays a vital role in the fight against infection (Shi et al., 2017). Gasdermin D (GSDMD) is a key effector molecule in the occurrence process of pyroptosis. Under stimulation with foreign substances, the intracellular pattern recognition receptor (nucleotide-binding domain leucine-rich repeat containing [NLR]) binds to the precursor of caspase-1 through the junction protein ASC and then forms a multi-protein complex to activate caspase-1. The activated caspase-1 then cleaves GSDMD to form a peptide containing the active domain of GSDM-NT, which induces the release of contents, cell membrane perforation and cell rupture, causing an inflammatory response. It also activates IL-1β and IL-18, which are released from the cell to recruit inflammatory cells and expand the inflammatory response (Broz et al., 2020; Liu et al., 2021). Pyroptosis may participate in the formation and development of tumors, and different tissues and genetic backgrounds of pyroptosis may have different effects on cancer. It can inhibit tumors but form a microenvironment suitable for the growth of tumor cells and then promote tumor growth (Xia et al., 2019). Studies have shown that pyroptosis can impact tumor cell proliferation, invasion and metastasis and further affect the cancer prognosis (Al Mamun et al., 2021). The expression of GSDMD in gastric cancer cells is lower than that in non-cancer cells, and the low expression of GSDMD promotes the proliferation of gastric cancer cells (Fang et al., 2020). Abnormally up-regulated GSDMB can also enhance the growth and invasive ability of bladder cancer cells (He et al., 2021). In addition, pyroptosis regulates the tumor immune microenvironment (TIME) and is involved in the body’s immune response to tumors (Xi et al., 2019; Zhang et al., 2020). It has been proven that tumor pyroptosis can enhance tumor immunogenicity by attracting more anti-tumor lymphocytes and reconstruct the local or systemic anti-tumor immunity by reversing the immunosuppressive microenvironment around tumor cells (Tan et al., 2021). Therefore, ‘inducing tumor pyroptosis’ is considered a potential cancer treatment strategy. Interestingly, lncRNAs are also mediators of cancer pyroptosis (Chen et al., 2020; Tang et al., 2021). However, the clinical significance of most pyroptosis-related (PR) lncRNAs has not been clearly investigated.

With the deepening of research in pyroptosis, an increasing number of PR genes have been identified. A PR signature has also been identified in various types of tumors, such as ovarian cancer (Ye et al., 2021), gastric cancer (Shao et al., 2021) and lung adenocarcinoma (Lin et al., 2021). Moreover, PR genes signatures have been established to predict the prognosis of patients with BLCA (Chen et al., 2021; Fu and Wang, 2022). Several studies have recently suggested that PR long non-coding RNAs (lncRNAs) may also participate in the formation and development of tumors. miRNA-214 was reported to inhibit the occurrence of glioma cells by directly targeting caspase-1 (Jiang et al., 2017). LncRNA GAS5 overexpression may also induce caspase-1 upregulation and promote pyroptosis in ovarian cancer cells (Li et al., 2018). At present, PR lncRNAs signatures also have been gradually developed in tumor research to predict the TIME changes and prognosis of tumor patients. Fada et al. (Xia et al., 2021) developed a 15 prognostic PR lncRNAs risk model to predict colon adenocarcinoma patients’ prognosis and TIME changes; Similar models have been developed in other tumor types, such as hepatocellular carcinoma (Wu et al., 2021) and kidney renal clear cell carcinoma (Tang et al., 2021). However, at present, few published PR lncRNAs signatures can be used to predict TIME changes and prognosis in patients with BLCA. The role and prognostic value of PR lncRNAs in BLCA have not been clarified.

In the present study, we comprehensively evaluated the molecular characteristics of these PR genes in BLCA and then identified PR lncRNAs with prognostic value based on the expression of PR genes. We performed a consistent clustering analysis of BLCA patients according to the expression of prognosis-related PR lncRNAs and identified two clusters. Based on these findings, the least absolute shrinkage and selection operator (LASSO) regression was used to establish a PR lncRNA signature and calculate the risk score associated with the prognosis of patients with BLCA. We also evaluated the relationship between the risk score and TIME. This study assesses the link between pyroptosis and TIME in BLCA, as well as provides a new reference to predict the prognosis of BLCA patients and identify personalized treatment strategies.

## Materials and Methods

### Acquisition of Data From Patients With Bladder Cancer

We obtained BLCA transcriptome, gene mutation data and clinical data from the TCGA database (https://portal.gdc.cancer.gov/). The mRNA and lncRNA expression profile data were derived from 414 BLCA tissues and 19 normal tissues, and gene mutation data samples were derived from 411 BLCA tissues. Gene copy number variation data were obtained from the UCSC database (https://xenabrowser.net/datapages/), including 413 BLCA samples. The clinical data are shown in Supplementary Table S1, we extracted clinical data from 412 patients. Samples without complete clinical information will be excluded in the subsequent clinical correlation analysis.

### Analyses of Molecular Characteristics of Pyroptosis-Related Genes in Bladder Cancer

The 33 PR genes were shown in Supplementary Table S2 **,** and these genes have been proved to be associated with pyroptosis in previously published studies (Man and Kanneganti, 2015; Wang and Yin, 2017; Karki and Kanneganti, 2019; Xia et al., 2019; Chen et al., 2021). We extracted the expression data of 33 PR genes from the BLCA transcriptome data. Using the limma package for the differential expression analysis, we extracted the copy number variation data of 33 PR genes from the data obtained from the UCSC database. We then counted the frequency of copy number variation of these genes in all samples. The RCircos package was used to visualize the change information of gene copy numbers. Similarly, we used the maftools package to analyze the mutation data of 33 PR genes from the mutation data obtained from the TCGA database and counted the mutation frequencies.

We used the Search Tool for Interaction Genes (STRING) database (https://string-db.org/cgi/input.pl) to construct PPI networks for differentially expressed PR genes and used the OncoLnc online analysis tool (http://www.oncolnc.org/) to perform a prognostic analysis of these genes. The OncoLnc tool can be used to analyze the correlation between mRNA, miRNA or lncRNA expression and the prognosis of patients with specific types of tumors based on the prognostic data of the TCGA database (Anaya, 2016).

### Identification of Pyroptosis-Related lncRNAs

We removed the samples with incomplete survival data, and 407 BLCA samples remained after merging with the PR lncRNA expression matrix. The co-expression method based on the expression of 33 PR genes was used to identify PR lncRNAs. A total of 812 PR lncRNAs were identified according to the criteria |correlation coefficient| > 0.4 and *p* < 0.01. The Igraph package was used to visualize the co-expression network. A univariate Cox regression analysis was performed to screen prognosis-related PR lncRNAs at *p* < 0.05.

### Analyzing the Correlation Between Tumor Clusters and Clinical Features

The ConsensusClusterPlus packet is an algorithm that can identify cluster members and their number in datasets (such as microarray gene expression profiles) (Wilkerson and Hayes, 2010). A consistent clustering analysis was used to determine the optimal number of clusters (*k*) and verify the clustering rationality by a resampling-based approach to assess the stability of the clusters. We used this package to perform a consistent clustering analysis based on the prognosis-related PR lncRNA expression matrix and then performed a prognostic correlation analysis of BLCA clusters. The degree of immune cell infiltration in BLCA was evaluated using the CIBERSORT algorithm (Newman et al., 2015). The results of the correlation analysis between the BLCA clusters and immune cell infiltration were considered significant at *p* < 0.05.

### Construction of Pyroptosis-Related lncRNA Signature

The BLCA patients were randomly divided into training and testing groups in a 1:1 ratio using the caret R package. The expression matrix of PR lncRNAs was combined with the prognosis data of the patients. A LASSO regression analysis was used to develop a PR lncRNA signature in the training group. The testing and entire groups were used to verify the established signature. The risk score of each BLCA patient was calculated according to the following formula:

Risk score = coefficient (lncRNA_1_) × expression (lncRNA_1_) + coefficient (lncRNA_2_) × expression (lncRNA_2_) + coefficient (lncRNA_3_) × expression (lncRNA_3_) + … + coefficient (lncRNA_n_) × expression (lncRNA_n_).

The BLCA patients in all groups were then identified as high- and low-risk patients based on the median risk score obtained from the training group.

### Analyzing the Prognostic Efficacy of the lncRNA Signature in Bladder Cancer

To determine whether or not the prognosis of the signature was independent of other clinical variables, univariate Cox and multivariate Cox regression analyses were used to calculate the values of the risk and other clinical features in predicting the prognosis of patients. The time-dependent receiver operating characteristic (ROC) curve was plotted using the survROC package. The area under the curve (AUC) at one, three and 5 years was calculated to determine the accuracy and specificity of the signature in predicting the prognosis.

### Analyzing the Correlation Between Risk Score and Other Factors

We analyzed the correlation between the patients’ clinical characteristics (including age, gender, grade and stage), tumor clusters and risk score. The expression of tumor immune checkpoint genes (ICGs) PD-1, PD-L1, PD-L2, CTLA-4, LAG3, CD47, CD4, CD8A and IDO1 in BLCA was obtained from the expression profile. The correlation between the risk score and ICGs was then analyzed. The principal component analysis (PCA) of risk in all BLCA patients was performed using the Rtsne R package to determine whether or not the signature could distinguish between high- and low-risk patients based on the expression of eight lncRNAs.

### A Gene Set Enrichment Analysis and Gene Enrichment Analysis

To understand the pathways that differ between the two clusters of BLCA in this study, a GSEA analysis among BLCA clusters was performed using the GSEA 4.1.0 software program, and the results of the pathway analysis were considered significant at a false discovery rate (FDR) of <0.05. To understand the functions and pathways that may be involved in differentially expressed genes between high- and low-risk BLCA, the samples were divided into high- and low-risk groups and then subjected to a gene differential expression analysis. The screening criteria for differentially expressed genes (DEGs) were FDR <0.05 and |log fold change (FC)| > 1. After obtaining DEGs, the DAVID 6.8 database (https://david.ncifcrf.gov/) was used to perform GO and KEGG analyses. All analysis results were considered significant at FDR <0.05.

### Statistical Analysis

Kaplan-Meier method was used to analyze the prognosis, and the Log rank test was used to determine the difference. The correlation between the two variables was tested by Spearman correlation analysis. Wilcoxon test was used to analyze the differences between high- and low-risk groups. The results of the above statistical analysis were considered significant at *p* < 0.05. Statistical analyses were performed using R software (version 4.1.2).

## Results

### Molecular Characterization of Pyroptosis-Related Genes and Identification of Pyroptosis-Related lncRNAs in Bladder Cancer

**FIGURE 1:**
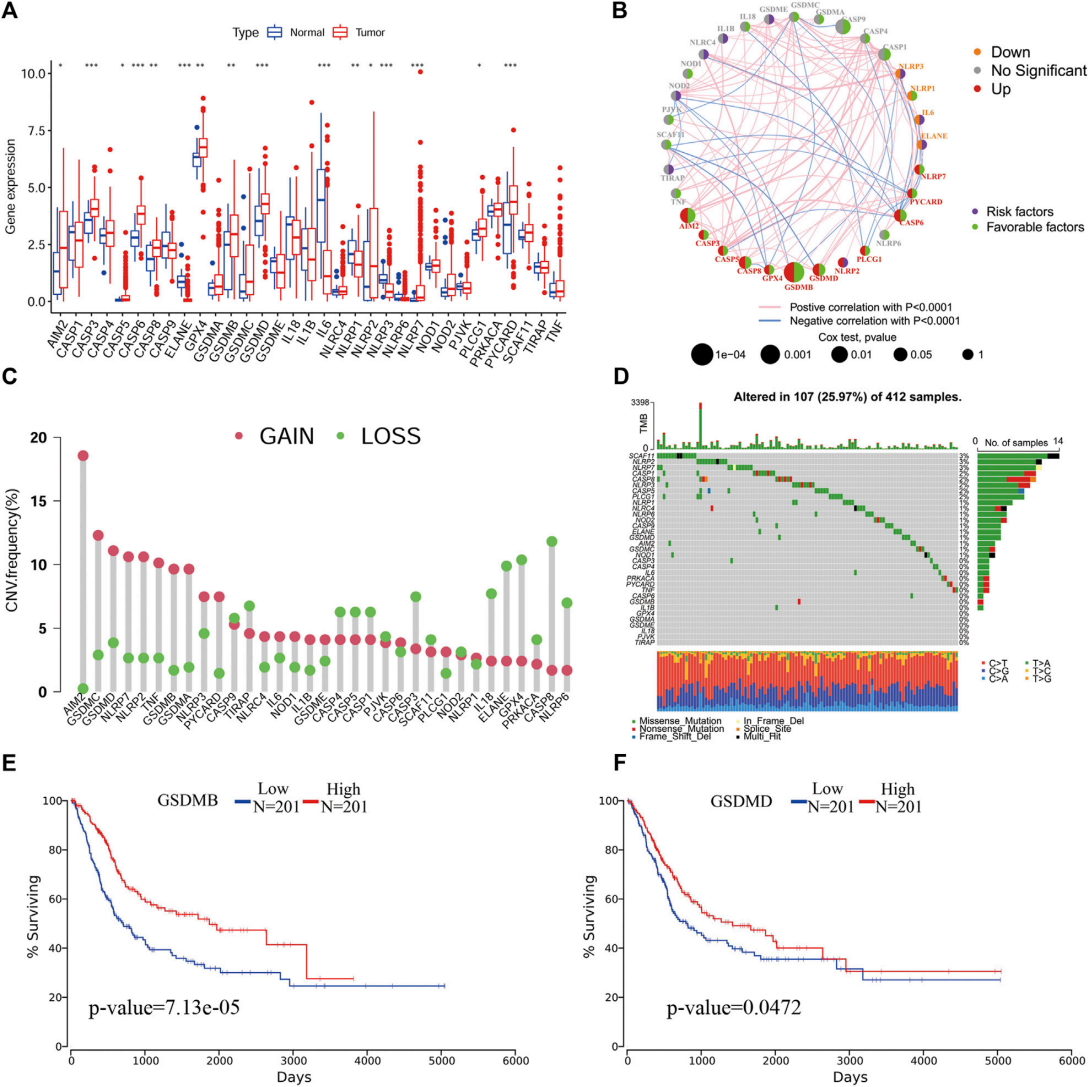
An analysis of the molecular characteristics of PR genes in BLCA. **(A)**, An expression analysis of PR genes in tumor and normal tissues *p* < 0.05(*), *p* < 0.01(**) and *p* < 0.001(***). **(B)**, Co-expression and univariate Cox regression analyses of PR genes in BLCA. **(C)** CNV analysis of PR genes in BLCA. **(D)** Mutation frequency analysis of PR genes in BLCA. **(E)** Kaplan-Meier survival analysis of GSDMB in BLCA. **(F)** Kaplan-Meier survival analysis of GSDMD in BLCA.

We extracted the expression data of 33 PR genes and analyzed the differences in the expression between normal and tumor tissues. We found that ELANE, IL6, NLRP1 and NLRP3 had a low differential expression in BLCA; however, AIM2, CASP3, CASP5, CASP6, CASP8, GPX4, GSDMB, GSDMD, NLRP2, NLRP7, PLCG1 and PYCARD had a high differential expression in BLCA (Figure 1A). The univariate Cox regression analysis results showed that GSDMB, CASP9, AIM2, CASP6, CASP8, CASP1 and GSDMD were significantly correlated with the prognosis and were protective factors (Figure 1B). The copy number variation analysis results showed that the copy number changes were consistent with their expression (Figure 1C), with the main copy number changes of AIM2, GSDMC, GSDMD, NLRP7 and NLRP2 showing amplication (gain), and these genes were also highly expressed in BLCA. A mutation analysis identified the three genes (SCAF11, NLRP2 and NLRP7) with the highest mutation rates (Figure 1D).

To clarify the relationships between the roles of pyroptosis genes, we performed a PPI network analysis. We found that PYCARD had the most network nodes, suggesting a possible crucial regulatory role of PYCARD in BLCA (Supplementary Figures S1A-S1B). A Kaplan-Meier survival analysis showed that the expression of GSDMB and GSDMD was significantly correlated with the survival of patients, and the prognosis of patients with a high expression was better than that of patients with a low expression (Figures 1E,F).

According to the criteria |correlation coefficient| > 0.4 and *p* < 0.01, a total of 812 PR lncRNAs were identified from the TCGA BLCA expression profile data, and the co-expression network of PR genes/lncRNAs was plotted (Supplementary Figure S1C). The prognosis-related PR lncRNAs were screened using a univariate Cox regression analysis, and 194 prognosis-related PR lncRNAs were obtained (Supplementary Figure S1D). These prognosis-related PR lncRNAs were identified for subsequent research.

### Results of Consistent Clustering Analysis of BLCA Based on Pyroptosis-Related lncRNAs

**FIGURE 2:**
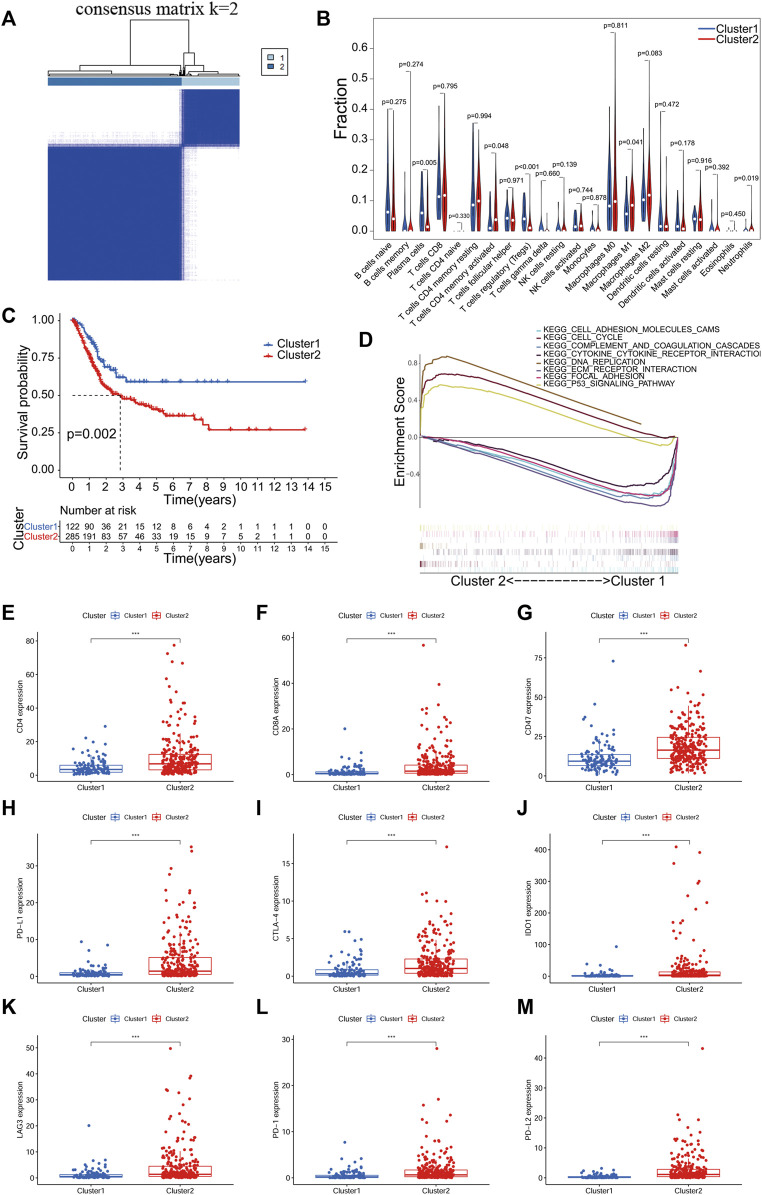
Consistent clustering analysis based on PR lncRNA of BLCA. **(A)**, The TCGA BLCA cohort divided into two clusters at *k* = 2. **(B)**, An analysis of the relationship between clusters of BLCA and immune cell infiltration. **(C)** Kaplan-Meier survival analysis of patients with two clusters of BLCA. **(D)** Gene set enrichment analysis (GSEA) predicted potential functions and pathways between the two clusters. **(E-M)**, Expression analysis of immune checkpoint genes in two clusters of BLCA. *p* < 0.05(*), *p* < 0.01(**) and *p* < 0.001(***).

A consensus clustering algorithm was used to classify groups of BLCA patients based on the expression of prognosis-related PR lncRNAs. The *k* = 2-9 cumulative distribution function (CDF) representing the clustering counts. *k* = 2 was determined as the optimal clustering parameter based on the similarity of the expression of prognosis-related PR lncRNAs and the ratio of the fuzzy clustering metric. The 407 BLCA patients with complete survival information were divided into 2 clusters: cluster 1 (n = 122) and cluster 2 (n = 285) (Figure 2A; Supplementary Table S3).

The infiltration level of 23 immune cell subtypes in each sample of BLCA was calculated using the CIBERSORT algorithm. The correlation analysis results between BLCA subtypes and infiltration level of immune cells showed significant differences in T cells CD4^+^ memory activated, T cells regulatory (Tregs), Plasma cells, Macrophages M1 and Neutrophils between different clusters (*p* < 0.05, Figure 2B). The overall survival of both clusters was calculated by the Kaplan-Meier method, and cluster one had a better prognosis than cluster 2 (*p* = 0.002, Figure 2C). In the GSEA analysis, we used FDR <0.05 as a filter and found that mainly the following pathways were activated between the two clusters: cell adhesion molecules cams, cell cycle, complement and coagulation cascades, cytokine-cytokine receptor interaction, DNA replication, ECM receptor interaction, focal adhesion and the p53 signaling pathway (Figure 2D).

In addition, we also analyzed the expression of ICGs among different clusters. The expression of all ICGs in cluster two was significantly higher than in cluster 1 (Figures 2E-M). It means that patients in cluster two are more likely to benefit from immunotargeted therapy.

### Development of a PR lncRNA Signature

**FIGURE 3:**
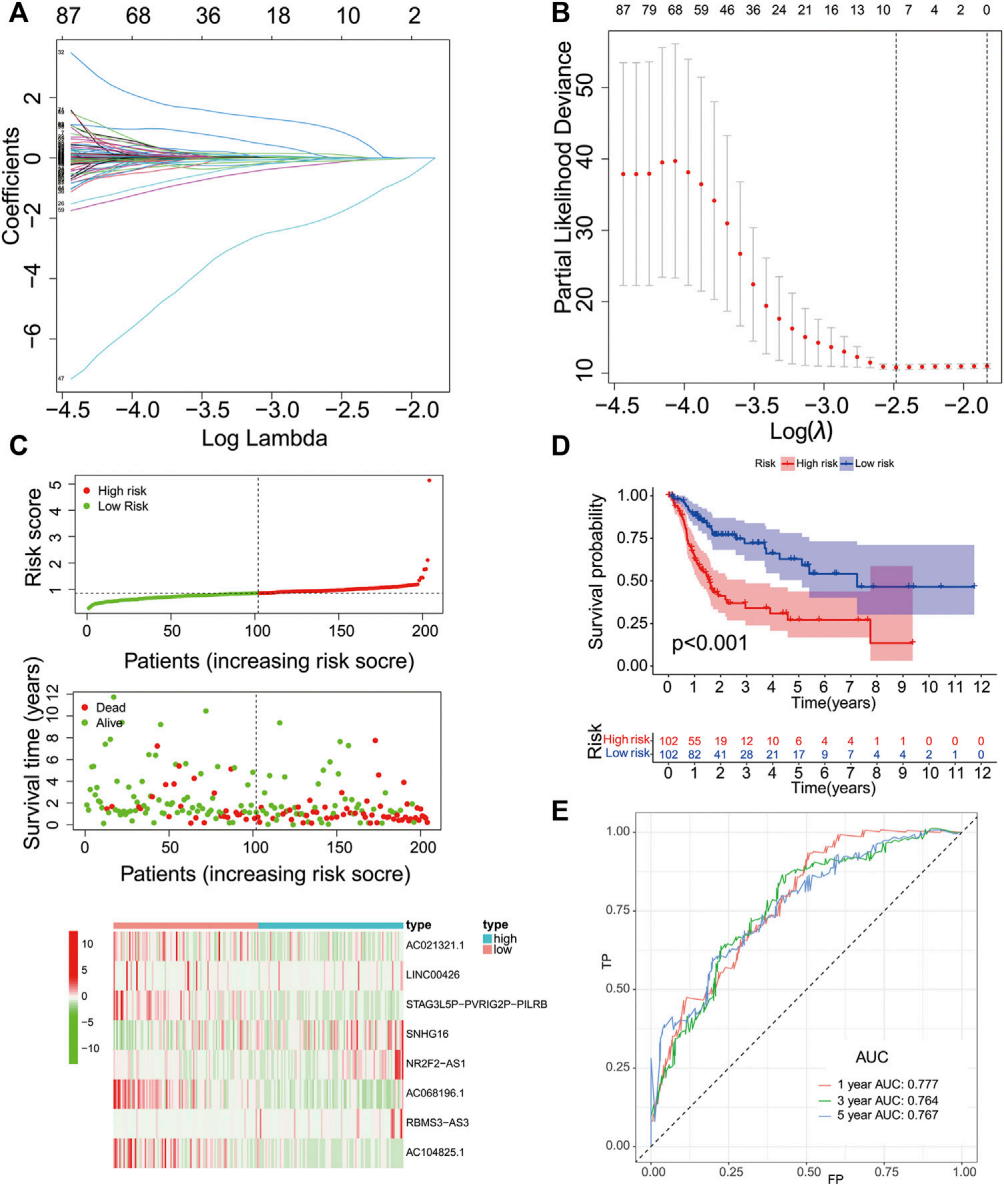
Construction of the PR lncRNA signature in the training group. **(A-B)**, The adjustment parameter (λ) selected in the LASSO model was cross-validated by a factor of 10 of the minimum criterion. **(C)**, The survival status and lncRNA expression heat map. **(D)**, An analysis of the overall survival of high- and low-risk patients in the training group. **(E)**, ROC curves of sensitivity and specificity of the signature for predicting the prognosis.

We then evaluated the reliability of PR lncRNAs for predicting the prognosis of patients. The BLCA patients were randomly divided into training (n = 204) and testing groups (n = 203). Eight significant lncRNAs were identified in the training group using a LASSO regression analysis: AC021321.1, LINC00426, STAG3L5P-PVRIG2P-PILRB, SNHG16, NR2F2-AS1, AC068196.1, RBMS3-AS3 and AC104825.1. The corresponding coefficient for each lncRNA was then obtained (Figures 3A,B). Risk scores were calculated for the training, testing and entire groups, as follows:

Risk score = -0.006334916 × expr (AC021321.1) - 0.123481702 × expr (LINC00426) - 0.095912859 × expr (STAG3L5P-PVRIG2P-PILRB) + 0.0163094 × expr (SNHG16) + 0.835268684 × expr (NR2F2-AS1) - 1.751229658 × expr (AC068196.1) + 0.095237679 × expr (RBMS3-AS3) - 0.042537256 × expr (AC104825.1).

The lncRNAs with positive coefficients in the formula are risk factors (SNHG16, NR2F2-AS1 and RBMS3-AS3), while the lncRNAs with negative coefficients are protective factors (AC021321.1, LINC00426, STAG3L5P-PVRIG2P-PILRB, AC068196.1 and AC104825.1).

The training group’s median risk score (0.8531) was used as the cut-off value, and patients were identified as low- and high-risk patients based on this cut-off value. The results in Figure 3C revealed that the patients with a high risk might have a poor prognosis. The OS analysis of the two groups showed that the OS of the high-risk group was significantly lower than that of the low-risk group (*p* < 0.001, Figure 3D). We used a time-dependent ROC curve to test the sensitivity and specificity of the diagnostic risk characteristics. In the training group, the AUC for predicting the patient survival at 1 year was 0.777, the AUC for predicting the survival at 3 years was 0.764, and the AUC for predicting the survival at 5 years was 0.767 (Figure 3E).

### Validation of the Signature in Other Groups

**FIGURE 4:**
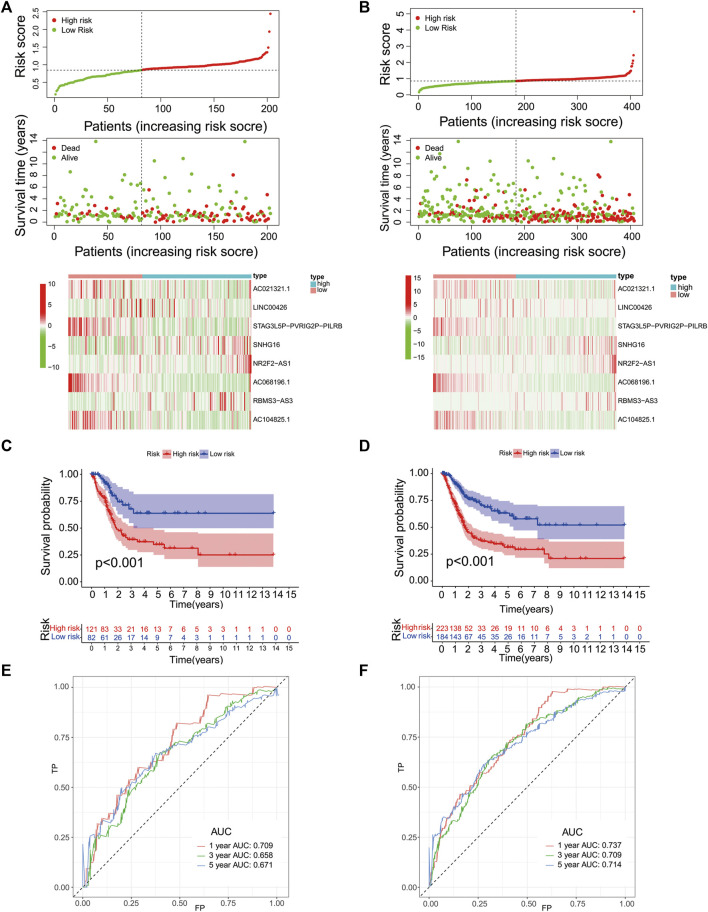
Performance validation of the eight-PR-lncRNA signature. **(A)**, A heat map of the survival status and lncRNA expression in high- and low-risk patients in the testing group. **(B)**, A heat map of the survival status and lncRNA expression in high- and low-risk patients in the entire group. **(C)**, An analysis of the overall survival of high- and low-risk patients in the testing group. **(D)**, An analysis of the overall survival of high- and low-risk patients in the entire group. **(E)**, An assessment of the sensitivity and specificity of the prognostic prediction of the eight-PR-lncRNA signature in the testing group. **(F)**, An assessment of the sensitivity and specificity of the prognostic prediction of the eight-PR-lncRNA signature in the entire group.

We then validated the predictive efficacy of the eight-PR-lncRNA signature in the testing and entire groups. The patients in these two groups were identified as high- and low-risk patients using the same methods. Figure 4A and Figure 4B show the relationship between the risk score and survival status in the two groups, respectively, and all results were consistent with the training group. Figures 4C-D show the prognostic differences between the high- and low-risk patients in the testing and entire groups, respectively. These results were also consistent with the training group. The overall survival of the high-risk group was significantly lower than that in the low-risk group (*p* < 0.001). The time-dependent ROC curve in the testing group is shown in Figure 4E, and the time-dependent ROC curve in the entire group is shown in Figure 4F; all of them obtained an ideal AUC value.

**FIGURE 5:**
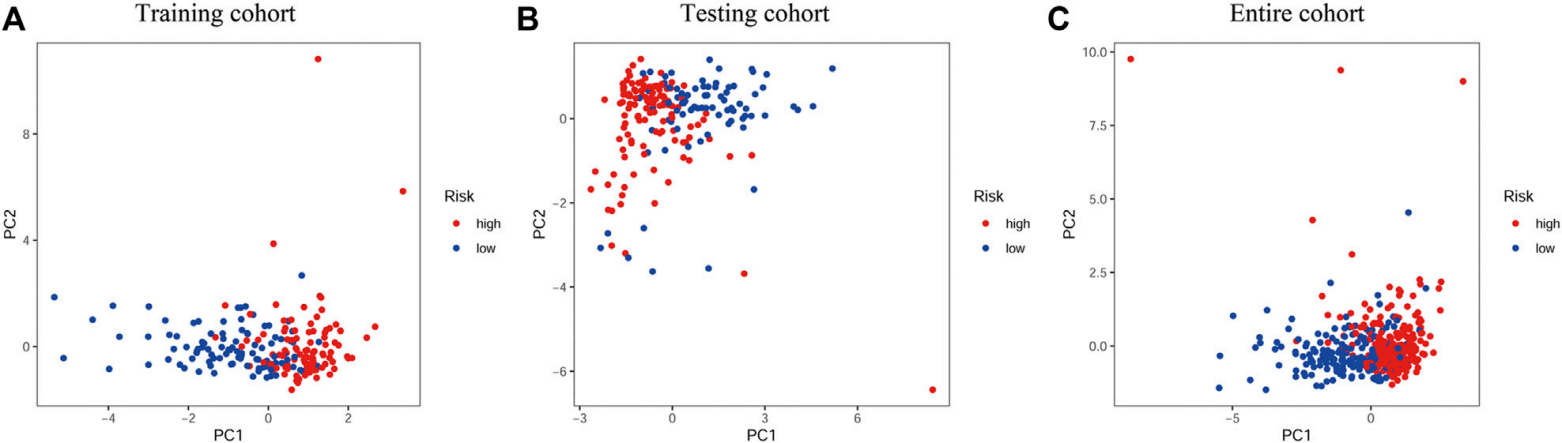
PCA analysis of the different distribution patterns of eight PR lncRNAs on genome-wide expression profiles. **(A)**, Training cohort. **(B)**, Testing cohort. **(C)**, Entire cohort.

We used a PCA analysis to examine the distribution patterns of the eight PR lncRNAs based on the expression profiles of all BLCA patients. The PCA analysis results suggested that the eight-PR-lncRNA signature could divide BLCA patients into high- and low-risk populations (Figures 5A–C).

### Correlation Between the Eight-PR-lncRNA Signature and Clinical Features

**FIGURE 6:**
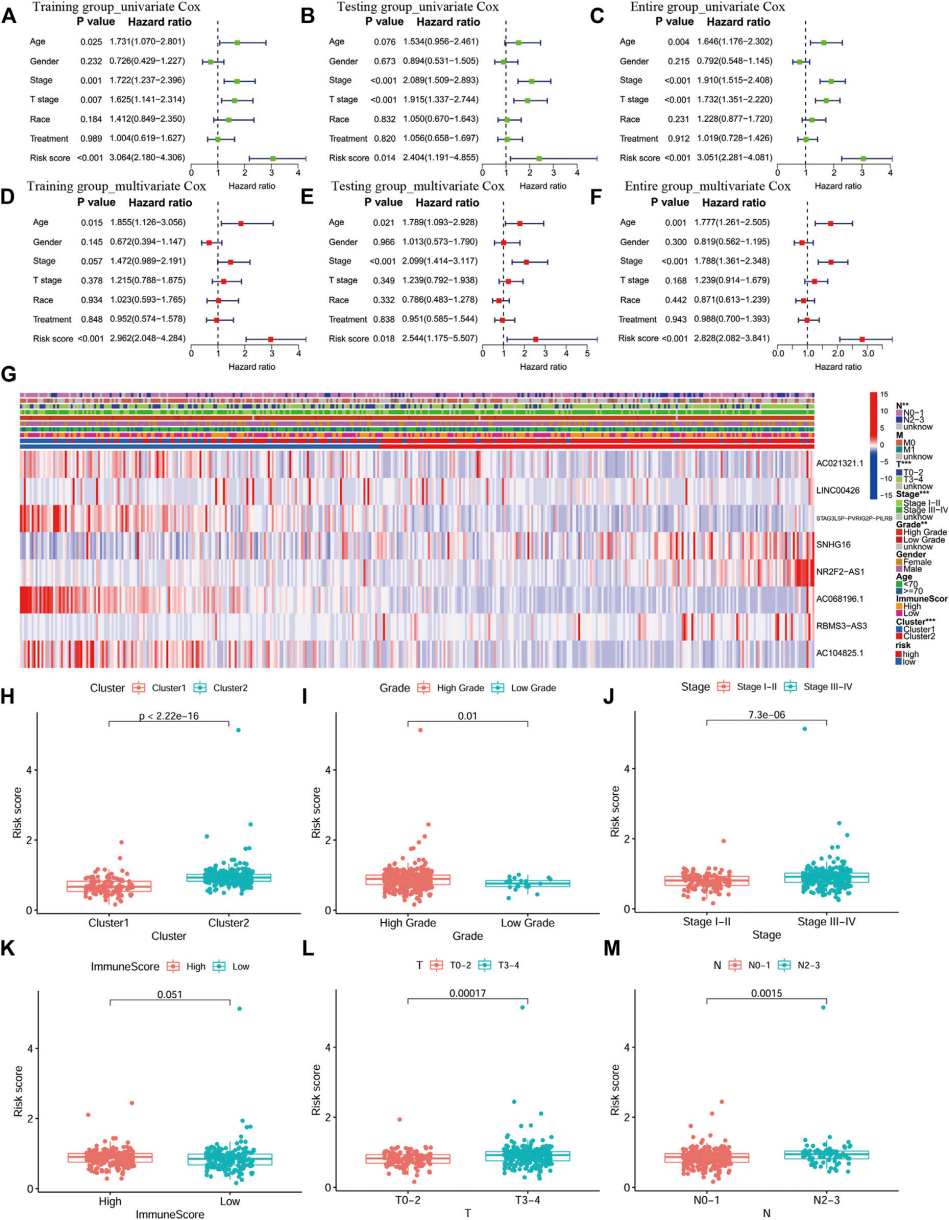
An independent prognostic analysis of the eight-PR-lncRNA signature and a correlation analysis between the risk score and clinical characteristics. **(A)** Univariate Cox regression analysis in the training group. **(B)**, Multivariate Cox regression analysis in the training group. **(C)** Univariate Cox regression analysis in the testing group. **(D)** Multivariate Cox regression analysis in the testing group. **(E)** Univariate Cox regression analysis in the entire group. **(F)** Multivariate Cox regression analysis in the entire group. **(G)** Heat map of the lncRNA expression and clinicopathological features in high- and low-risk patients. *p* < 0.05 (*), *p* < 0.01 (**) and *p* < 0.001 (***). **(H)**, The distribution of risk score in the two groups of consistent clustering results. **(I-J)**, The distribution of risk score by grade and stage of BLCA. **(K)**, The distribution of risk score in the ImmuneScore-high and ImmuneScore-low groups. **(L)**, The distribution of risk score by T stage. **(M)**, The distribution of risk score by N stage.

The univariate and multivariate Cox analyses were used to analyze the performance of the signature in the training, testing and entire groups to identify independent factors for the overall survival (OS). The results of the three groups showed that risk was an independent factor associated with a poor prognosis in BLCA patients (*p* < 0.05; Figures 6A–F). The same analysis was performed in the entire group. The heat map visualized the differences in the expression of eight selected PR lncRNAs between the high- and low-risk groups and annotated clinical information (Figure 6G). Cluster two had a significantly higher risk than cluster 1 (*p* < 0.001, Figure 6H), consistent with the previous OS analysis results. In addition, the risk score of a high grade for BLCA was significantly higher than that of low-grade disease (Figure 6I). The same results were also obtained for the stage (*p* < 0.001, Figure 6J), T stage (*p* < 0.001, Figure 6L) and N stage (*p* = 0.0015, Figure 6M). However, there was no significant difference between the high- and low-ImmuneScore groups (*p* = 0.051, Figure 6K).

**FIGURE 7:**
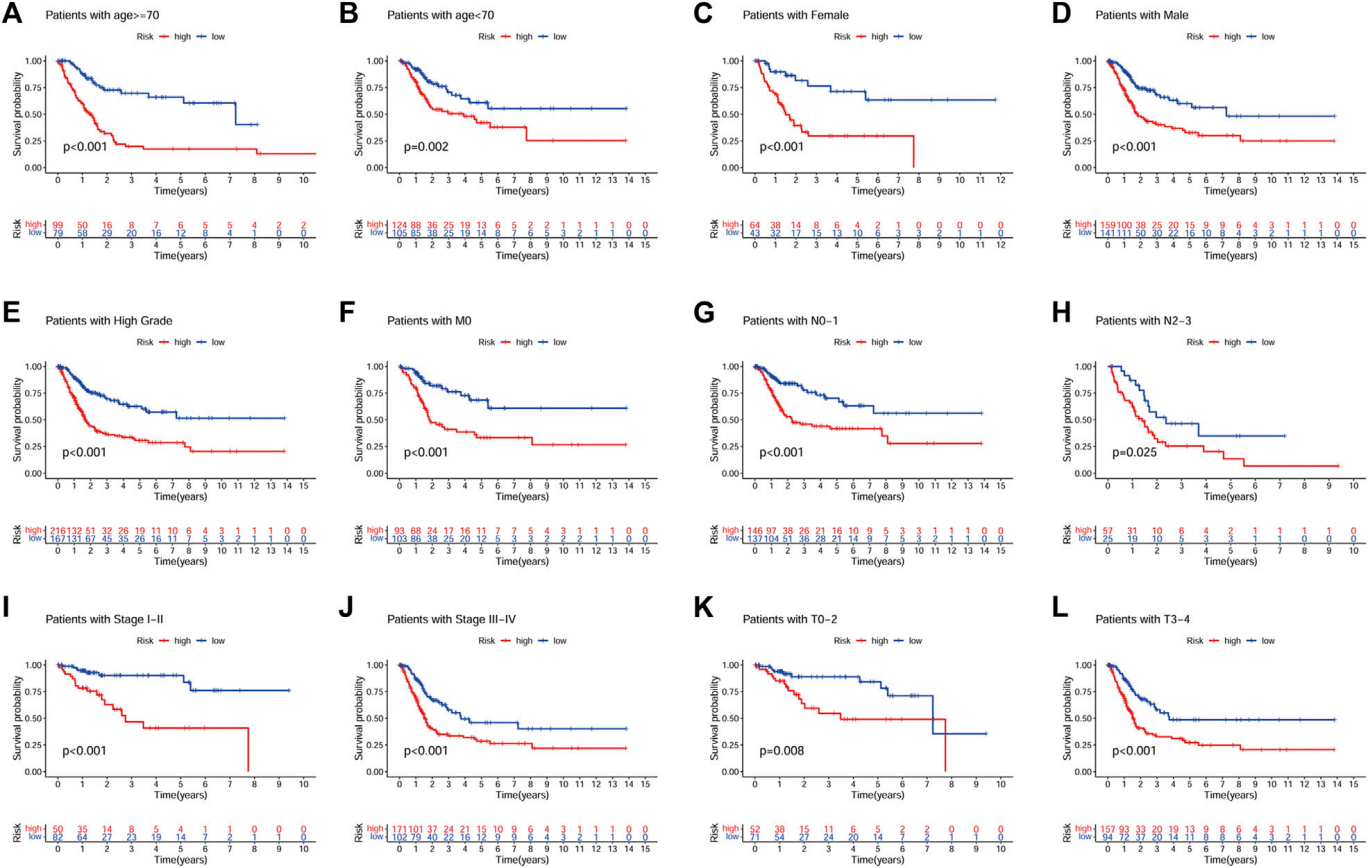
Prognostic analysis of high- and low-risk patients in different clinical characteristics subgroups. **(A)**, Age ≥ 70. **(B)**, Age < 70. **(C)**, Female. **(D)**, Male. **(E)**, High grade. **(F)**, M0. **(G)**, N0-N1. **(H)**, N2-N3. **(I)**, Stage I-II. **(J)**, Stage III-IV. **(K)**, T0-T2. **(L)**, T3-T4.

A prognostic analysis of high- and low-risk patients in specific clinical characteristics subgroups (age, gender, grade, stage, T, M and N) showed that the prognosis of high-risk patients was poor in all clinical characteristics subgroups, except for the low-grade and M1 subgroups (Figure 7).

### Correlation Between Risk Score and Tumor Immunity

**FIGURE 8:**
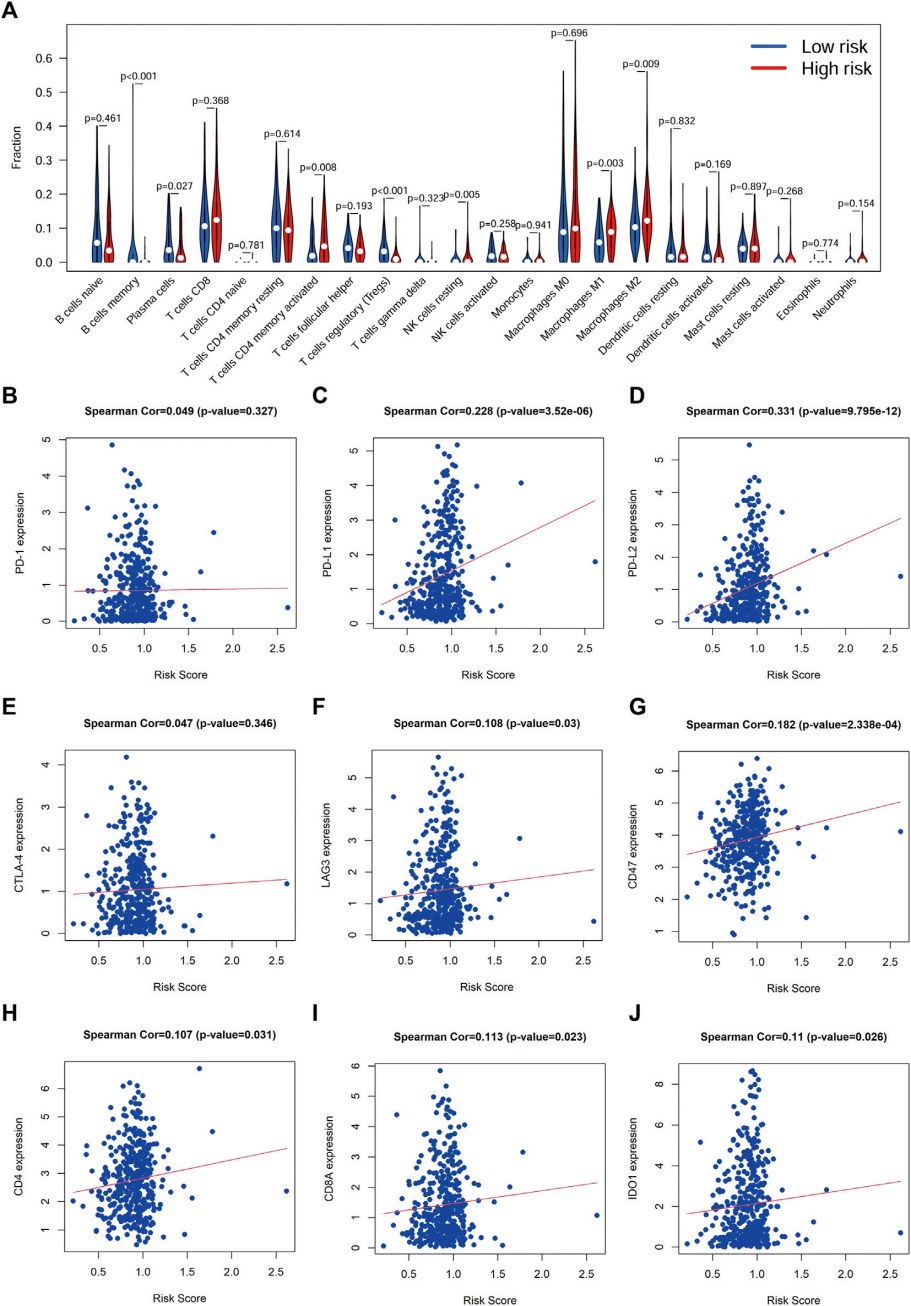
Correlation between risk score and immune cell infiltration and ICGs **(A)**, Correlation analysis between risk score and immune cell infiltration. **(B-J)**, Correlation analysis between the risk score and immune checkpoint genes.

To understand the relationship between the risk score and the TIME of BLCA, we analyzed the correlation between the risk score and the infiltration level of 23 immune cell subtypes, with the results shown in Figure 8A. Interestingly, there was a degree of heterogeneity in the levels of B-cell, T-cell, NK-cell and Dendritic cell infiltration between the high-risk and low-risk groups. We also examined the correlation between the risk score and the expression of ICGs, and the results showed that the risk score was significantly positively correlated with multiple ICGs (*p* < 0.05; Figures 8B–J).

### Expression and Function Analysis of the Eight lncRNAs in the Signature

**FIGURE 9:**
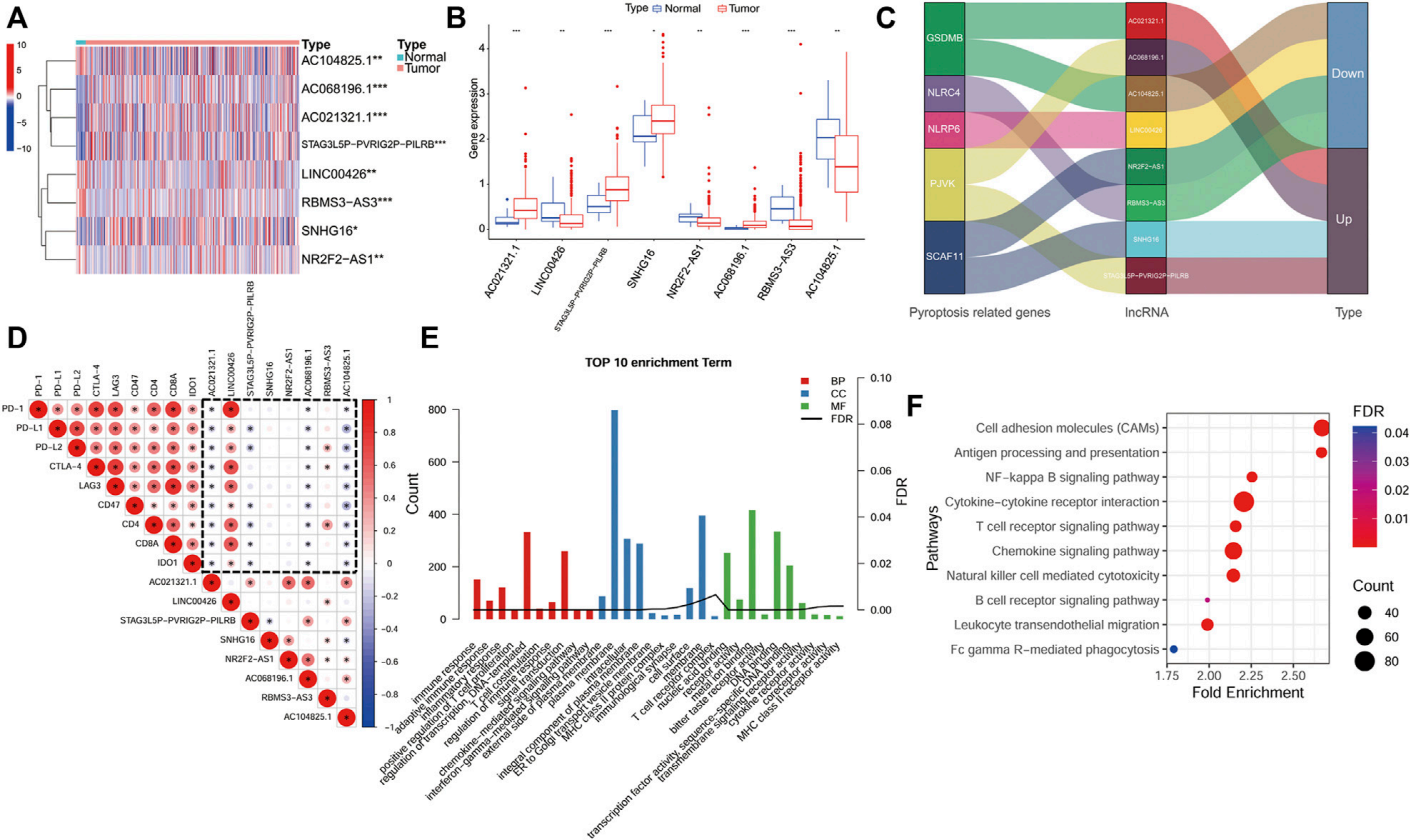
Expression and functional analyses of lncRNAs in the signature. **(A,B)**, An expression analysis of the eight lncRNAs in BLCA tissues and normal tissues *p* < 0.05(*), *p* < 0.01(**) and *p* < 0.001(***). **(C)**, The regulatory relationship between the lncRNAs in the signature and PR genes. **(D)**, An analysis of the correlation between the immune checkpoint genes and the eight lncRNA expression (dotted frame). **(E)**, Results of a GO enrichment analysis of the protein-coding genes (PCGs). **(F)**, Results of a KEGG analysis of PCGs.

We also evaluated the expression of eight lncRNAs in BLCA. The results showed that the expression of LINC00426, NR2F2-AS1, RBMS3-AS3 and AC104825.1 in BLCA tissue was lower than that in normal tissues, while the expression of AC021321.1, STAG3L5P-PVRIG2P-PILRB, SNHG16 and AC068196.1 in BLCA tissue was higher than that in normal tissues (Figures 9A,B). Figure 9C demonstrates the regulatory relationship between these lncRNAs and PR genes. In addition, we also analyzed the expression correlation between the ICGs and lncRNAs in the signature. We found that AC021321.1, AC104825.1, AC068196.1 had a negative correlation with all ICGs, while LINC00426 had a positive correlation with all ICGs (*p* < 0.05; Figure 9D). To understand the possible function and mechanism of these eight lncRNAs in BLCA, we used a co-expression method to find the protein-coding genes (PCGs) of these eight lncRNAs, and the screening criteria were |Pearson correlation coefficient| > 0.4 and *p* < 0.001 (Gao et al., 2019). A total of 3141 PCGs were obtained, and these PCGs were submitted to the functional enrichment analysis using the DAVID database with FDR <0.05. GO enrichment results showed that these PCGs were mainly enriched in human immune response functions, such as the immune response (BP), inflammatory response (BP), T cell costimulation, regulation of immune response (BP), MHC class II protein complex (CC), T cell receptor complex (CC), immunological synapse (CC), cytokine receptor activity (MF) and MHC class II receptor activity (MF) (Figure 9E). The KEGG pathway enrichment analysis showed that these genes were also mainly enriched in immunomodulatory pathways, such as cytokine−cytokine receptor interaction, T cell receptor signaling pathway, B cell receptor signaling pathway and natural killer cell mediated cytotoxicity (Figure 9F).

### Results of a Functional Analysis Between High- and Low-Risk Groups and Construction of a Nomogram

We also analyzed the functions and pathways involved in the DEGs in high- and low-risk groups. According to the screening criteria |logFC| > 1 and FDR <0.05, a total of 1017 DEGs were screened. Immune-related functions were found in the GO analysis results, including inflammatory response (BP) (Supplementary Figure S2A), and the KEGG enrichment analysis also identified immune-related pathways, such as cytokine-cytokine receptor interaction (Supplementary Figure S2B).

**FIGURE 10:**
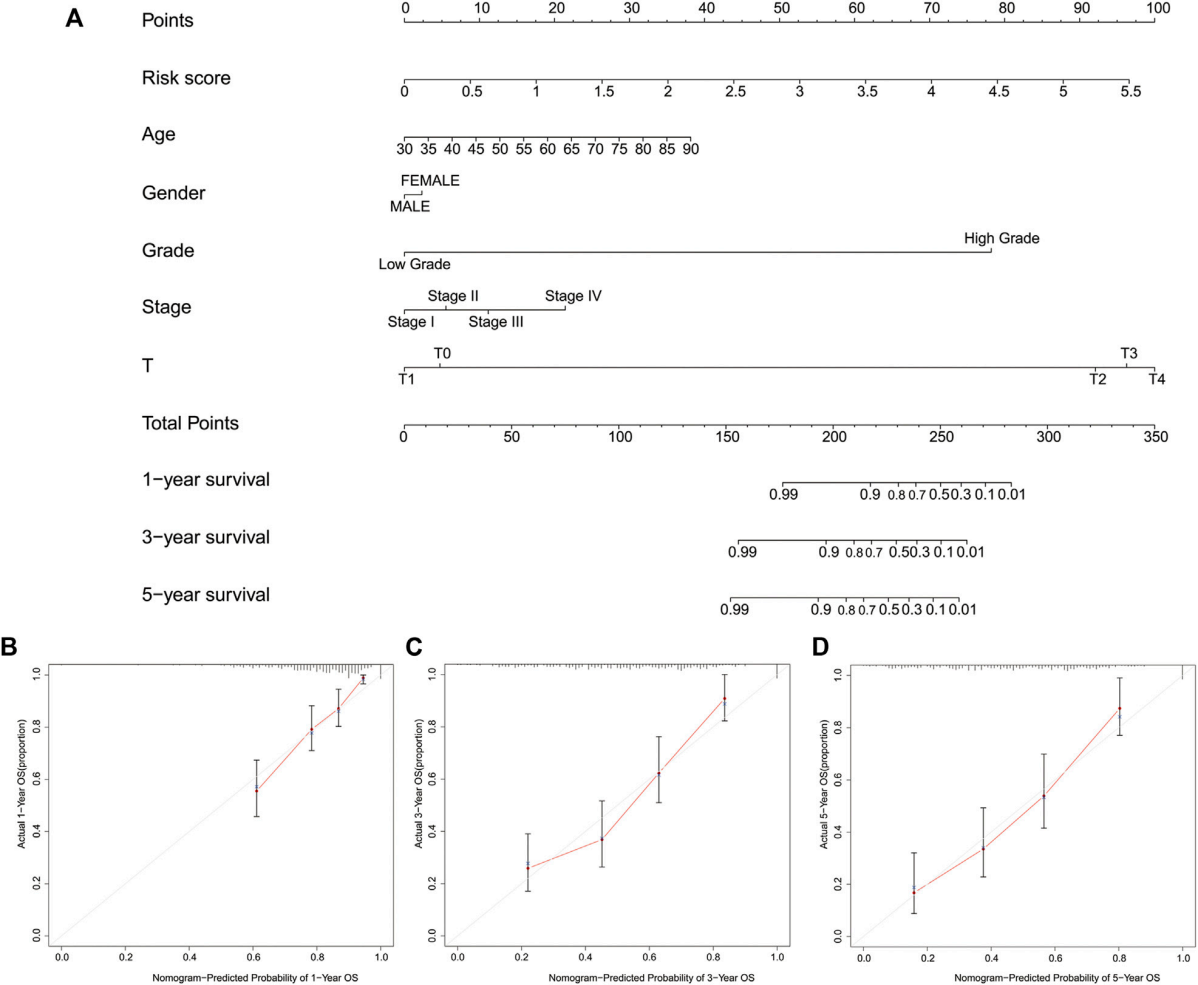
Construction of a nomogram predicting the one-, three- and 5-year overall survival. **(A)**, A nomogram of the probability of predicting prognosis. **(B-D)**, The calibration plot of the nomogram.

To facilitate the clinical use of our signature to predict the prognosis of BLCA patients, we also developed a nomogram including risk classification and clinical risk characteristics to predict the one-, three- and 5-year OS (Figure 10A). The risk scores of the prognostic signature had superior predictive power to other clinical factors. The calibration plots showed that the observation and prediction rates of the OS had ideal consistency (Figures 10B–D).

## Discussion

BLCA is a tumor of the urinary system with a high incidence. Due to its complex pathogenesis, there are several different genetic subtypes of tumors, and these subtypes may have different therapeutic responses to the same treatment. If not correctly treated, BLCA can have a high morbidity and mortality (Kamat et al., 2016).

Cell death is a common topic in life science. Tumor cells have the ability to escape cell death contributes to the origin of tumors. This ability also plays a crucial role in acquiring treatment resistance, developing recurrence and metastasizing (Hanahan and Weinberg, 2011). Pyroptosis is a type of programmed cell death in inflammation mediated by GSDM (Lu et al., 2021). Our findings found that patients with high GSDMD and GSDMB expression had a better prognosis, and the results of the GSDMD analysis were consistent with previously published studies (Fang et al., 2020). However, the better prognosis of patients with high GSDMB expression seems to contradict previous studies finding that high expression of this gene in bladder cancer promotes tumor cell proliferation (He et al., 2021). Currently, there is controversy regarding the role of GSDMB in tumors. GSDMB is also involved in pyroptosis, it can promote atypical pyroptosis by enhancing the activity of caspase-4 and has the function of inhibiting the proliferation of tumor cells (Li et al., 2020). It is still not clear whether the GSDMB protein cleaved by caspase-3/-6/-7 is involved in pyroptosis. Our results further confirm that the role of GSDMB in tumorigenesis is controversial, indicating that GSDMB has great research value in future research.

Human genome sequencing data has shown that most RNA transcripts of non-protein-coding origin are transcribed from more than 90% of the human genome (Mattick and Makunin, 2006). With further research, more studies have shown that lncRNAs also play an essential role in the development and malignant progression of BLCA (Li et al., 2020). It has been reported that lncRNAs are involved in the pathological processes of various diseases through direct or indirect actions on proteins related to the pyroptosis signaling pathway (He et al., 2020). The release of cytokines produced by pyroptosis changes the TIME and promotes the growth of tumors by evading immune surveillance (Loveless et al., 2021). However, at present, there are few PR lncRNA signatures have been developed for BLCA.

We identified 812 PR lncRNAs based on the expression of 33 PR genes, and 194 prognosis-related PR lncRNAs were screened by a univariate Cox regression analysis. The BLCA cohort was then divided into two clusters based on the prognosis-related PR lncRNAs expression using consistent clustering. We found that the degree of infiltration of some immune cells differed significantly among clusters. The expression of the ICGs in cluster two was considerably higher than that in cluster 1, suggesting that patients in cluster two were more likely to have tumor immune escape and benefit from ICI therapy. In addition, the OS of cluster one was better than that of cluster 2, and the tumor grade of cluster one was also lower than that of cluster 2. The results of a GSEA analysis suggested that the following pathways were related to tumor development and metastasis: cell adhesion molecules cams (Cohen et al., 1997; Zhou et al., 2021), cell cycle (Li et al., 2021), cytokine-cytokine receptor interaction (Tang et al., 2020), focal adhesion (Tong et al., 2022) and p53 signaling pathway (Jiao et al., 2020). These results suggest a potential relationship between PR lncRNAs and the progression of BLCA. Consistent cluster analyses based on the PR lncRNA expression may help improve the efficacy of immunotherapy for BLCA.

We next applied LASSO regression to the training group to construct eight-PR-lncRNA signature (including AC021321.1, LINC00426, STAG3L5P-PVRIG2P-PILRB, SNHG16, NR2F2-AS1, AC068196.1, RBMS3-AS3 and AC104825.1). LncRNAs play an integral role in human epigenetic regulatory mechanisms. They participate in biological processes through epigenetic, transcriptional, post-transcriptional and translation regulatory targets, including cell growth, metastasis and apoptosis (Mirzaei et al., 2021, 2022). Their dysfunction is closely related to tumorigenesis (Han et al., 2020; Shigeyasu et al., 2020). Previous studies have shown that LINC00426 and SNHG16 can promote tumor development and participate in the regulation of TIME (Chen et al., 2020; Tao et al., 2020). LINC00426 and SNHG16 play an important role in the occurrence and development of tumors (Li et al., 2020; Wan et al., 2022). NR2F2-AS1 can down-regulate the expression of PDCD4 and inhibit the development of gastric cancer through competitive binding with miR-320b, it can also inhibit miR-494 methylation to regulate oral squamous cell carcinoma cells proliferation (Liang et al., 2022; Luo et al., 2022). Overexpression of RBMS3-AS3 inhibits cell proliferation, migration, invasion, angiogenesis and tumorigenicity of prostate cancer by up-regulating VASH1 (Jiang et al., 2020). The published evidence mentioned above suggests that these PR lncRNAs we identified are indeed associated with tumor development. While other lncRNAs AC021321.1, STAG3L5P-PVRIG2P-PILRB, AC068196.1 and AC104825.1 in our signature have not been reported in any published tumor studies, all were studied for the first time in our study. Our findings may provide evidence for future studies of these lncRNAs.

In the verification group, the signature also showed the same predictive performance as the training group. The OS analysis results indicated that an eight-PR-lncRNA signature could predict the survival rate of BLCA patients to some extent. We also found that risk score from eight-PR-lncRNA signature was an independent prognosis factor for BLCA patients. Patients with high-grade disease had a higher risk score than those with low-grade disease, and the same results were obtained between clusters 1 and 2, which was consistent with the conclusion that the OS of cluster one was better than that of cluster 2. The results of the risk score and ICGs correlation analysis suggested that patients with a high risk were more likely to experience tumor immune escape and benefit more from ICI therapy than others (Gao et al., 2020). Our results were consistent with the published results that pyroptosis can also increase the efficiency of tumor immunotherapy by recruiting immune cells and activating the immune system, its anti-tumor effect is also closely related to multiple ICGs (such as PD-1 or PD-L1) (Li et al., 2021).

To understand the possible function of these eight lncRNAs in BLCA, we used the co-expression method to find the co-expressed PCGs of the eight lncRNAs. The results of PCGs functional enrichment suggested that these eight lncRNAs may have immunomodulatory functions. Similarly, the enrichment analysis of genes that were differentially expressed between the high- and low-risk groups also found immune-related processes and pathways, such as inflammatory response (BP) and cytokine-cytokine receptor interaction (KEGG) (Bao and Cao, 2016). Furthermore, we also developed a nomogram containing risk classification and clinical risk characteristics to facilitate the clinical development and utilization of our findings (Iasonos et al., 2008). All these findings establish a close association between PR lncRNAs and the prognosis of BLCA patients as well as changes in TIME. The shortcoming of this study was the lack of lncRNA expression data from other sources for external validation, this is because we cannot find a suitable dataset containing these eight lncRNAs probes in other source datasets. Therefore, further external validation is needed to verify the reliability of the signature, and experimental validation of the role of these lncRNAs in BLCA cells should also be performed in the future.

## Conclusions

Our study systematically evaluated the molecular biological characteristics and prognostic value of PR genes/lncRNAs in BLCA and identified an eight-PR-lncRNA signature (including AC021321.1, LINC00426, STAG3L5P-PVRIG2P-PILRB, SNHG16, NR2F2-AS1, AC068196.1, RBMS3-AS3 and AC104825.1) related to the prognosis of BLCA patients. We also analyzed the role of this signature in the TIME and its potential regulatory mechanisms, which provides an essential basis for future studies concerning the relationship between PR lncRNAs and BLCA immunity. Our findings will also help identify novel prognostic biomarkers and therapeutic targets for BLCA.

## Data Availability

The original contributions presented in the study are included in the article/Supplementary Material, further inquiries can be directed to the corresponding author.
