# Supplementary figures and images for: Genome-wide Exploration of a Pyroptosis-Related Long Non-Coding RNA Signature Associated With the Prognosis and Immune Response in Patients With Bladder Cancer
Source: Front Genet. 2022 Apr 27;13:865204. doi: 10.3389/fgene.2022.865204 (PMC9091201; doi:10.3389/fgene.2022.865204)

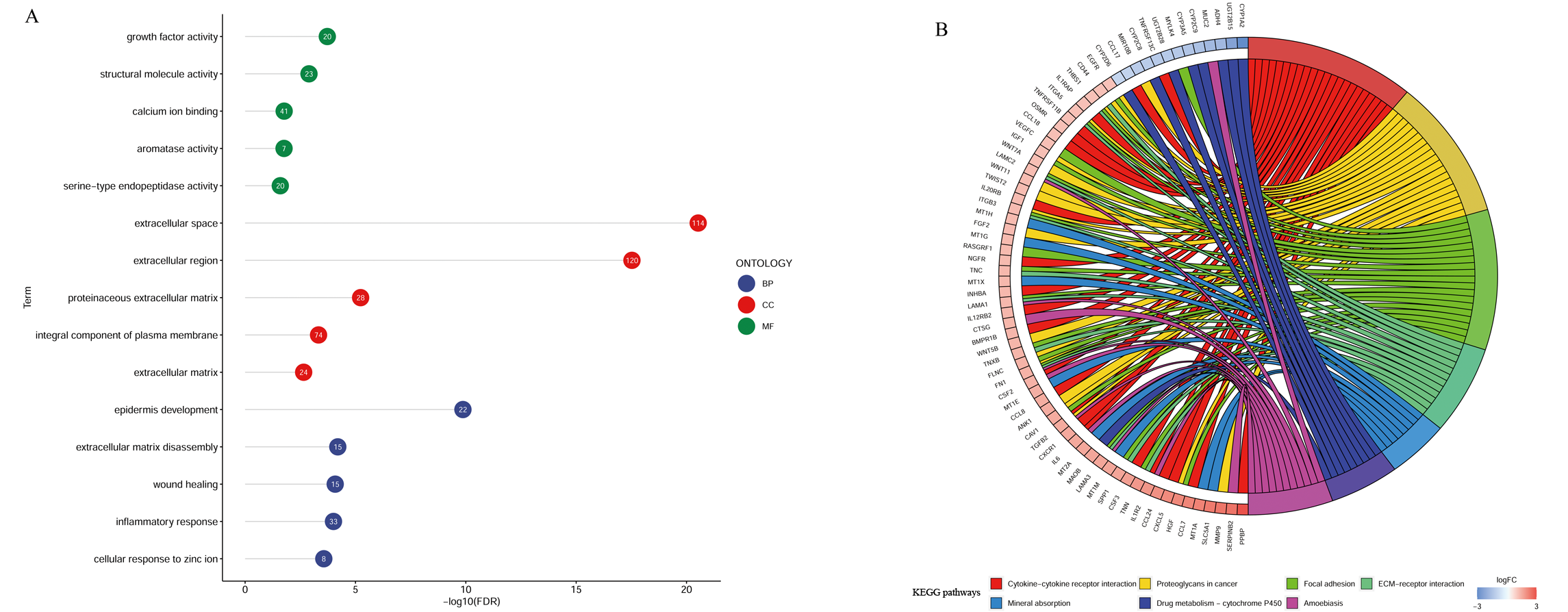

Supplement: Supplementary file 3 [file Image2.tif]

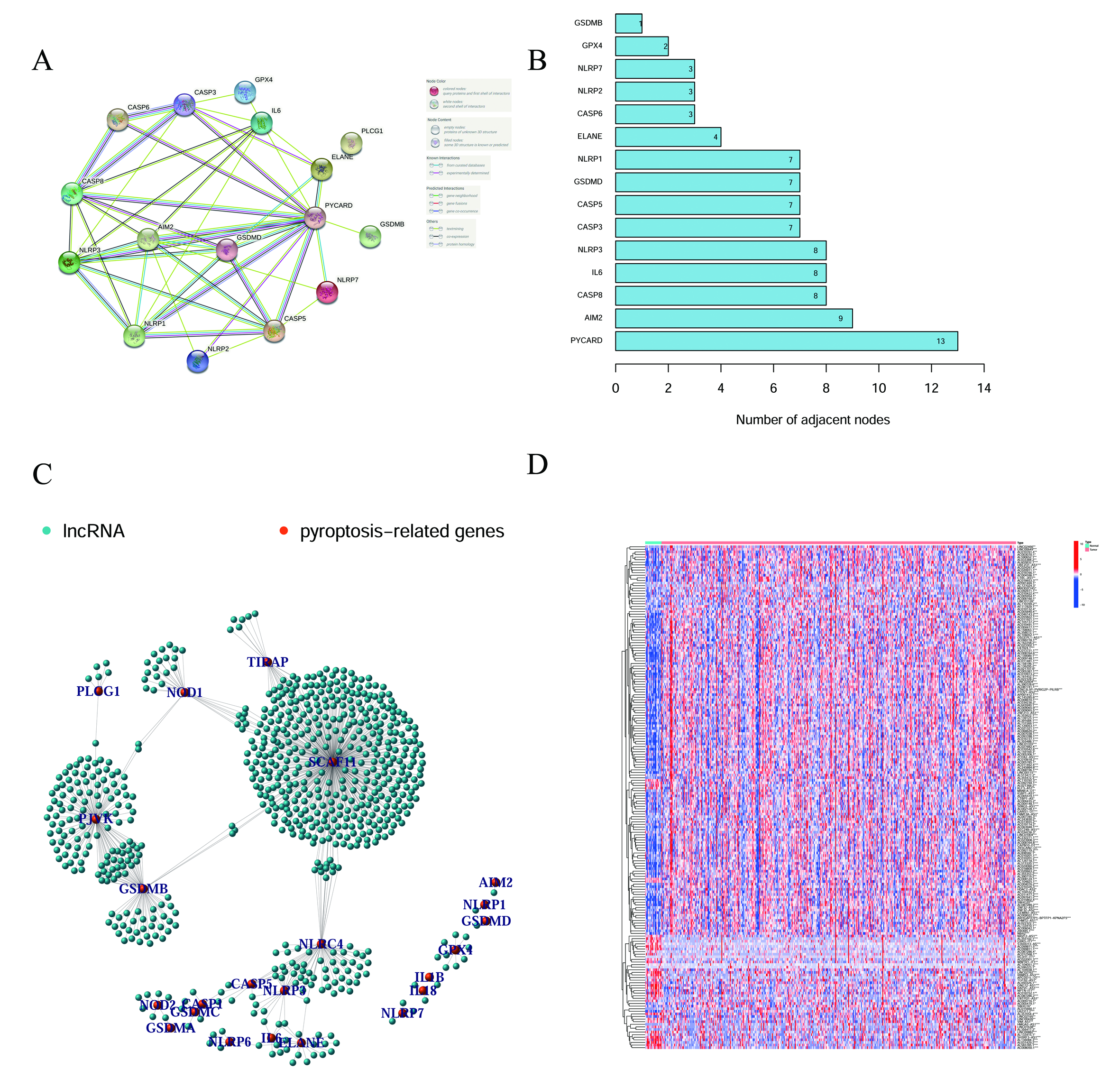

Supplement: Supplementary file 4 [file Image1.TIF]
