# Supplementary material for: Genome-wide Exploration of a Pyroptosis-Related Long Non-Coding RNA Signature Associated With the Prognosis and Immune Response in Patients With Bladder Cancer
Source: Front Genet. 2022 Apr 27;13:865204. doi: 10.3389/fgene.2022.865204 (PMC9091201; doi:10.3389/fgene.2022.865204)
Supplement: Supplementary file 6 [file DataSheet1.docx]

**Supplementary Figure Captions**

**Figure S1** Protein-protein interaction network (PPI) network analysis of PR genes and identification of PR lncRNAs in BLCA. A, PPI network analysis of PR genes; B, Ranking of gene nodes from PPI network; C, co-expression network of PR genes/lncRNAs in BLCA. D, heat map of prognosis-related PR lncRNAs expression in BLCA.

**Figure S2** GO functional (A) & KEGG pathway enrichment analysis (B) of differentially expressed genes between high- and low-risk groups.

**Supplementary Table Captions**

**Table S1** The clinical data of BLCA from the TCGA database

**Table S1** List of 33 pyroptosis-related genes (PR genes)

**Table S3** Two clusters of BLCA samples
